# The deubiquitinase BRCC3 increases the stability of ZEB1 and promotes the proliferation and metastasis of triple-negative breast cancer cells: BRCC3 promotes the proliferation and metastasis of TNBC

**DOI:** 10.3724/abbs.2024005

**Published:** 2024-03-07

**Authors:** Qidi Huang, Shurong Zheng, Huayan Gu, Zhi Yang, Yiqiao Lu, Xia Yu, Guilong Guo

**Affiliations:** 1 Department of Breast Surgery the First Affiliated Hospital of Wenzhou Medical University Wenzhou 325000 China; 2 Department of Pathology the First Affiliated Hospital of Wenzhou Medical University Wenzhou 325000 China

**Keywords:** BRCC3, ZEB1, epithelial-mesenchymal transition, triple negative breast cancer

## Abstract

Triple negative breast cancer (TNBC) has a high recurrence rate, metastasis rate and mortality rate. The aim of this study is to identify new targets for the treatment of TNBC. Clinical samples are used for screening deubiquitinating enzymes (DUBs). MDA-MB-231 cells and a TNBC mouse model are used for *in vitro* and *in vivo* experiments, respectively. Western blot analysis is used to detect the protein expressions of DUBs, zinc finger E-box binding homeobox 1 (ZEB1), and epithelial-mesenchymal transition (EMT)-related markers. Colony formation and transwell assays are used to detect the proliferation, migration and invasion of TNBC cells. Wound healing assay is used to detect the mobility of TNBC cells. Immunoprecipitation assay is used to detect the interaction between breast cancer susceptibility gene 1/2-containing complex subunit 3 (BRCC3) and *Z*EB1. ZEB1 ubiquitination levels, protein stability, and protein degradation are also examined. Pathological changes in the lung tissues are detected via HE staining. Our results show a significant positive correlation between the expressions of BRCC3 and ZEB1 in clinical TNBC tissues. Interference with BRCC3 inhibits TNBC cell proliferation, migration, invasion and EMT. BRCC3 interacts with ZEB1 and interferes with BRCC3 to inhibit ZEB1 expression by increasing ZEB1 ubiquitination. Interference with BRCC3 inhibits TNBC cell tumorigenesis and lung metastasis *in vivo*. In all, this study demonstrates that BRCC3 can increase the stability of ZEB1, upregulate ZEB1 expression, and promote the proliferation, migration, invasion, EMT, and metastasis of TNBC cells, providing a new direction for cancer therapy.

## Introduction

Globally, breast cancer is one of the most common malignancies and the second leading cause of cancer death in women [1]. Triple-negative breast cancer (TNBC), which accounts for approximately 15% of all breast cancers, is highly aggressive and characterized by the absence of estrogen receptor (ER), progesterone receptor (PR), and human epidermal growth factor receptor 2 (HER2) [2]. Compared to other pathological types of breast cancers, TNBC tends to be associated with a younger age of onset, higher histologic grade, larger tumor volume, greater tendency toward recurrence and distant metastasis, and worse prognosis [ 3, 4]. Due to the lack of clear molecular markers, chemotherapy is currently the only effective systematic therapy for TNBC. Early-stage TNBC may still recur even if patients are in remission after chemotherapy, and the overall survival (OS) of patients with metastatic lesions is only 13‒18 months [5]. Current therapeutic regimens fail to improve the survival rate of TNBC patients, and there is an urgent need to explore new molecular targets for TNBC treatment.

It is well known that epithelial-mesenchymal transition (EMT) promotes tumor invasion and metastasis and is associated with the recurrence and metastasis of malignant tumors [ 6– 8]. The zinc finger E-box binding homeobox (ZEB) family (ZEB1 and ZEB2) comprises important transcription factors that regulate EMT. Many previous studies have identified a role of ZEB1 in the induction of EMT and metastasis in TNBC [ 9– 12]. Furthermore, ZEB1 expression is upregulated in TNBC tissues compared to adjacent normal tissues, and downregulation of ZEB1 inhibits cell proliferation, leading to the alleviation of cell migration and invasion [ 13, 14]. Therefore, ZEB1 plays a very important role in TNBC proliferation and metastasis, and inhibition of ZEB1 will become a research hotspot for TNBC treatment.

The ubiquitin-proteasome system (UPS) is a crucial pathway for intracellular protein degradation and is involved in a wide range of cellular activities, including apoptosis, cell cycle, and tumorigenesis [15]. Ubiquitination is a reversible process regulated by deubiquitinating enzymes (DUBs) which increase protein stability by removing ubiquitin from the substrate and preventing the degradation of the target protein via the UPS [16]. ZEB1 is regulated by DUBs, which ultimately leads to enhanced tumor invasion and metastatic capacity [ 17– 20]. In addition, recent studies have suggested that DUBs play key roles in the occurrence and development of TNBC. Therefore, DUBs may be potential targets for TNBC therapy, and an in-depth investigation of this mechanism is highly important for the treatment of TNBC.

In the present study, we identify BRCC3, a DUB that binds to ZEB1, but the role of BRCC3 in TNBC proliferation and metastasis has not yet been reported. The aim of this study was to investigate the role of BRCC3-mediated increase of ZEB1 stability in influencing the progression of TNBC.

## Materials and Methods

### Clinical samples

Twenty TNBC patients who underwent surgical treatment at the First Affiliated Hospital of Wenzhou Medical University (January 2021‒December 2021) were randomly selected. The patients had not undergone neoadjuvant therapy before surgery, and the diagnosis of TNBC was confirmed by pathology and immunohistochemistry in our Pathology Department. Tumor tissues and adjacent nontumor tissues approximately 5 cm away from the tumor tissue were paired. The patients provided signed informed consent, and the study was ethically approved by the Medical Ethics Committee of the First Affiliated Hospital of Wenzhou Medical University.

### Cell culture and transfection

Human mammary epithelial cell line (MCF-10A) and TNBC cell lines (MDA-MB-231, MDA-MB-453, MDA-MB-468, and BT-549) were purchased from American Tissue Culture Collection (ATCC; Manassas, USA). MCF-10A cells were cultured in DMEM/F12 complete medium (Gibco, Carlsbad, USA) supplemented with 5% horse serum (Sigma, St Louis, USA) and 10 μg/mL insulin (Procell Life Science & Technology Co. Ltd., Wuhan, China), 5 μg/mL hydrocortisone (Sangon Biotech, Shanghai, China), and 20 ng/mL epidermal growth factor (PeproTech, Waltham, USA) at 37°C with 5% CO _2_. TNBC cells were cultured in 1640 complete medium (Gibco) supplemented with 10% fetal bovine serum at 37°C with 5% CO _2_.

MDA-MB-231 and BT-549 cells in the logarithmic growth phase were inoculated into 6-well plates at 5×10 ^5^ cells/well and then cultured at 37°C with 5% CO _2_. MDA-MB-231 cells were divided into shRNA, shRNA-BRCC3, shRNA+vector, shRNA-BRCC3+vector and shRNA-BRCC3+ZEB1 groups. The BT-549 cells were divided into pcDNA and pcDNA-BRCC3 groups. When the cell confluence reached 70%, transient transfection of the cells in the experimental groups was performed using Lipofectamine 2000 (Invitrogen, Carlsbad, USA). After 5 h of cell transfection, the culture medium was replaced by culture medium, and the cells were cultured for an additional 48 h. Each group of cells was collected for subsequent experiments. The sequences of sense strand for shRNA-NC and shRNA-BRCC3 were as follows: shRNA: 5′-UCACUGCGCUCGAUGCAGUTT-3′ and shRNA-BRCC3: 5′-GUACUGGGUUUGUUACAGAUU-3′.

### Western blot analysis

Approximately 50 mg of tissue fragments were removed and rapidly ground by addition of a small amount of liquid nitrogen. Tissues or cells were collected, and RIPA lysis buffer was added to the cells on ice. Cell lysates were collected, centrifuged and subsequently measured using a BCA kit (Sigma). Fifty micrograms of protein sample was separated by sodium dodecyl sulfate-polyacrylamide gel electrophoresis, transferred to PVDF membranes (Millipore, Billerica, USA), blocked with 5% skim milk for 1 h and incubated overnight with primary antibodies as follows: anti-ZEB1 (ab203829, 1:500; Abcam, Cambridge, UK), anti-STAMBP (sc-398480, 1:1000; Santa Cruz Biotech, Santa Cruz, USA), anti-BAP1 (ab245391, 1:2000; Abcam), anti-UCHL1 (sc-271639, 1:1000; Santa Cruz Biotech), anti-USP9X (ab19879, 1:1000; Abcam), anti-BRCC3 (ab115172, 1:2000; Abcam), anti-E-cadherin (sc-8426, 1:1000; Santa Cruz Biotech), anti-vimentin (sc-6260, 1:1000; Santa Cruz Biotech), and anti-β-actin (ab227387, 1:5000; Abcam). The membrane was washed three times with TBST and incubated with an HRP-conjugated secondary antibody (1:3000; Cell Signaling Technology, Beverly, USA) for 30 min. After extensive wash, protein bands were visualized using ECL plus regent (Beyotime, Shanghai, China). ChemiDoc Image Lab software (Bio-Rad, Hercules, USA) was used for quantitative analysis. β-Actin was used as the loading control. The experiments were repeated three times.

### Immunofluorescence microscopy

MDA-MB-231 and BT-549 cells in the logarithmic growth phase were collected, washed three times with PBS and fixed with 4% paraformaldehyde (Beyotime) for 30 min. After being washed with PBS, MDA-MB-231 and BT-549 cells were treated with 0.5% Triton X-100+1% normal goat serum (Yeasen Biotechnology, Shanghai, China), and the cells were lysed and fixed on ice for 10 min. Next, MDA-MB-231 and BT-549 cells were blocked with 1% normal goat serum+PBS, incubated at 4°C for 2 h with primary antibodies as follows: anti-BRCC3 (ab62075, 20 μg/mL; Abcam), anti-ZEB1 (ab81972, 10 μg/mL; Abcam), anti-E-cadherin (ab227639, 1:100; Abcam), or anti-vimentin (ab16700, 1:1000; Abcam), and washed three times with 1% normal goat serum, followed by incubation with corresponding secondary antibodies [Cy3-labeled Donkey anti-Goat IgG (A0502, 1:500, Beyotime; red light); Dylight 488 labeled Goat anti-Rabbit IgG (ab96899; Abcam; green light)] for 1 h in the dark. After 3 times wash with PBS, the nuclei were stained with DAPI (Sigma), and the colocalization of BRCC3 and ZEB1 was observed under a fluorescence microscope.

### Real-time reverse transcription quantitative polymerase chain reaction (RT-qPCR)

The collected cells were subjected to RNA extraction using Trizol reagent (Invitrogen, Carlsbad, USA). Reverse transcription was performed using the PrimeScript RT kit (TaKaRa, Dalian, China). RT-qPCR was performed on an ABI 7500 PCR system (Foster City, USA) using the SYBR® Premix Ex TaqTM II kit (TaKaRa). The relative mRNA level of BRCC3 was detected by the 2 ^‒ΔΔCt^ method. *β-actin* was used as an internal reference. The primers used for the experiments were synthesized by Sangon Biotech (Shanghai, China) and the sequence information is shown in Table 1. All the experiments were repeated three times.

**Table:** **Table 1** Sequences of primers used for qPCR analysis

Gene	Transcript ID	Primer sequence (5′→3′)
*BRCC3*	NM_001018055.3	Forward: AGGAAGTAATGGGGCTGTGC
		Reverse: TCGATTCTCTCATACTCTGA
		ACTCT
*β-actin*	NM_001101.5	Forward: AGAAGGATTCCTATGTGGG
		CGAC
		Reverse: AGTACTTGCGCTCAGGAGGA

### Colony formation assay

A total of 2.5×10 ^6^ cells/well were inoculated in a 6-well plate, and adherent cells were treated according to the experimental methods. After digestion and resuspension, the cells were inoculated at 1×10 ^3^ cells/well in a new 6-well plate, and the culture was terminated when cell colonies appeared. The cells in each well were fixed with 4% paraformaldehyde (PFA) for 30 min, stained with 0.1% crystal violet for 30 min, washed, air-dried, and photographed. The number of colonies of more than 50 cells was counted. Three replicate wells were set up for each group.

### Transwell assay

The cells were starved with serum-free medium for 12 h, after which the cell concentration was adjusted to 5×10 ^4^ cells/mL. The cell suspension (200 μL) was placed in the upper chamber and serum-containing medium (600 μL) was added to the lower chamber, which was subsequently incubated at 37°C for 24 h. The chambers were gently removed, and the residual cells that had not penetrated the membrane were removed. The cells were fixed with 4% paraformaldehyde for 20 min and then stained with 1% crystal violet for 15 min. The migrated cells were observed and then counted under a microscope. The procedure for the cell invasion experiment was the same as above, but the transwell chambers were coated with Matrigel.

### Wound healing assay

After 48 h of transfection, the cells were collected by trypsin digestion and inoculated in 6-well plates at 1×10 ^6^ cells/well. After adhesion, the cells were scratched with a 200-μL pipette tip and washed with precooled phosphate buffer 3 times. Then, serum-free medium was added for routine culture. At 0 and 24 h, images were recorded using an inverted microscope. ImageJ software was used to calculate the rate of scratch healing as follows: rate of scratch healing=(the scratch width at 0 h‒the scratch width at 24 h)/the scratch width at 0 h×100%. Three replicate wells were set up for each group.

### Coimmunoprecipitation (IP) assay

MDA-MB-231 cells were transfected with the Flag-ZEB1 or Myc-BRCC3 plasmid for 48 h and then treated with MG132 (20 μM; Invitrogen). The precipitate was repeatedly washed 4–5 times by adding 0.5 mL of precooled NP40 (Beyotime) for 4 h. The cells were washed 3 times with phosphate-buffered saline, and then 500 μL of NP40 lysate containing protease inhibitor was added. The mixture was resuspended on ice for 20 min, followed by ultrasonic lysis and centrifugation at 4°C and 3000 *g* for 10 min. A total of 50 μL of supernatant was used as the input, and 30 μL of the corresponding antibody (anti-Flag or anti-Myc) was added, and the supernatant was incubated overnight at 4°C. Protein A+G agarose (Santa Cruz Biotech) was added, and the mixture was subsequently incubated at 4°C for 4 h. After centrifugation at 4°C and 3000 *g* for 3 min, the precipitate was repeatedly washed 4‒5 times with 0.5 mL of precooled NP40. Finally, loading buffer was added and the proteins were subject to western blot analysis using anti-ZEB1 (ab203829, 1:500; Abcam) and anti-BRCC3 (ab115172, 1:2000; Abcam) antibodies.

To examine the interaction between endogenous BRCC3 and ZEB1 in MDA-MB-231 cells, 20 μL of Protein A/G Agarose was mixed with 5 μL of anti-ZEB1 antibody (ab276129; Abcam) or anti-BRCC3 antibody (ab115172; Abcam) and incubated for 2 h at room temperature, after which the supernatant was added and incubated overnight at 4°C. IgG was used as a negative control. The next day, the supernatant was discarded, an appropriate amount of loading buffer was added, and 25 μg of total protein was subject to western blot analysis using anti-ZEB1 (ab203829, 1:500; Abcam) and anti-BRCC3 (ab115172, 1:2000; Abcam) antibodies.

### Ubiquitination assay

MDA-MB-231 cells were transfected with shRNA-BRCC3 or the negative control and were grouped into HA-ubiquitin (Ub), Flag-ZEB1+HA-Ub, Flag-ZEB1+shRNA+HA-Ub, or Flag-ZEB1+shRNA-BRCC3+HA-Ub groups. BT-549 cells were transfected with wild-type (WT) BRCC3 or mutant (Mut) BRCC3 and grouped into the following groups: Flag-ZEB1+HA-Ub, Flag-ZEB1+WT-BRCC3+HA-Ub, and Flag-ZEB1+Mut-BRCC3+HA-Ub. After MG132 (20 μM) was added, the cells were collected after 8 h and centrifuged at 4°C and 1600 *g* for 5 min. The supernatant was discarded, and the mixture was placed on ice. The cells were resuspended by adding 400 μL of HEPES buffer (Thermo Fisher, Waltham, USA), lysed by ultrasonication, and centrifuged at 4°C and 3000 *g* for 10 min. Then, 40 μL of supernatant was mixed with 2×loading buffer, and the expression of intracellular proteins was detected by western blot analysis using anti-ZEB1 antibody (ab203829, 1:500; Abcam). The remaining 360 μL of supernatant was added to 2 μL of anti-Flag antibody and incubated at 4°C for 4‒6 h. Then, 40–60 μL of protein A/G agarose was added and incubated at 4°C for 8–10 h. After centrifugation at 4°C and 1600 *g* for 3 min, supernatant was discarded, and the beads were mixed with 2× loading buffer and subject to western blot analysis using anti-ZEB1 antibody (ab203829, 1:500; Abcam).

### Cycloheximide chase assay

Cycloheximide (CHX, 10 μg/mL; Gibco) was added to MDA-MB-231 cells in the shRNA-BRCC3 or shRNA groups. Protein samples were collected at different time points (0, 1, 2, 3 and 4 h) after which western blot analysis was performed to detect ZEB1 protein expression.

### MG132 treatment

MDA-MB-231 cells in the shRNA-BRCC3 or shRNA groups were treated with or without 10 μM MG132 for 4 h. Protein samples were collected, and western blot analysis was subsequently performed to detect ZEB1 protein expression.

### Animal experiment

MDA-MB-231 cells expressing LV-shRNA, LV-shRNA-BRCC3 or LV-shRNA-BRCC3+LV-ZEB1 were prepared as a suspension of 3×10 ^6^ cells/mL. Two hundred microliters of the cell suspension was inoculated into the axilla of the right upper limb of BALB/c nude mice (4‒6 weeks old, female; Zhejiang Vital River Laboratory Animal Technology Co., Ltd., Jiaxing, China). Mice were divided into the control, LV-shRNA, LV-shRNA-BRCC3, and LV-shRNA-BRCC3+LV-ZEB1 groups (6 mice/group). Tumor diameters were measured regularly on a weekly basis using Vernier callipers, and tumor volumes were calculated. Mice were euthanized six weeks later, after which tumor tissues and lung tissues were excised. Animal experiments were approved by the Animal Experiments Ethics Committee of the First Affiliated Hospital of Wenzhou Medical University.

### HE staining

The lung tissues were cleaned with normal saline and fixed overnight with 4% paraformaldehyde. On the second day, paraffin embedding and sectioning were performed, and lung tissue sections were stained according to the instructions of the HE staining kit (Beyotime). After staining, the lung tissue sections were sealed, observed under a microscope, photographed and recorded.

### Immunohistochemistry assay

Specimens were serially sectioned (4 μm), deparaffinized, hydrated, and treated with 3% H _2_O _2_ to block endogenous peroxidase activity. After antigen retrieval in sodium citrate buffer (pH 9.0), sections were incubated overnight at 4°C with diluted primary antibodies as follows: anti-BRCC3 (ab62075, 2.5 μg/mL; Abcam), anti-ZEB1 (ab81972, 10 μg/mL; Abcam), anti-E-cadherin (ab227639, 1:100; Abcam), or anti-vimentin (ab16700, 1:200; Abcam). Then sections were incubated for 20 min with horseradish peroxidase (HRP)-labelled Goat anti-Rabbit IgG (ab205718, 1:2000; Abcam) or HRP-labeled Donkey anti-Goat IgG (A0181, 1:1000; Beyotime). Finally, sections were developed with DAB for 2 min, counterstained with hematoxylin for 1 min, and examined under a microscope after sealing [21]. PBS was used instead of the primary antibody as a negative control.

### Statistical analysis

SPSS 17.0 software was used for statistical analysis. Data are expressed as the mean ± standard deviation. Comparisons between two groups were performed by *t* test. Comparisons among multiple groups were analyzed by one-way ANOVA with Tukey′s or Bonferroni′s multiple comparison and two-way ANOVA with Bonferroni′s multiple comparison. A *P* value less than 0.05 indicated that the difference was statistically significant.

## Results

### Screening of ZEB1-associated DUBs in TNBC

**Figure 1:**
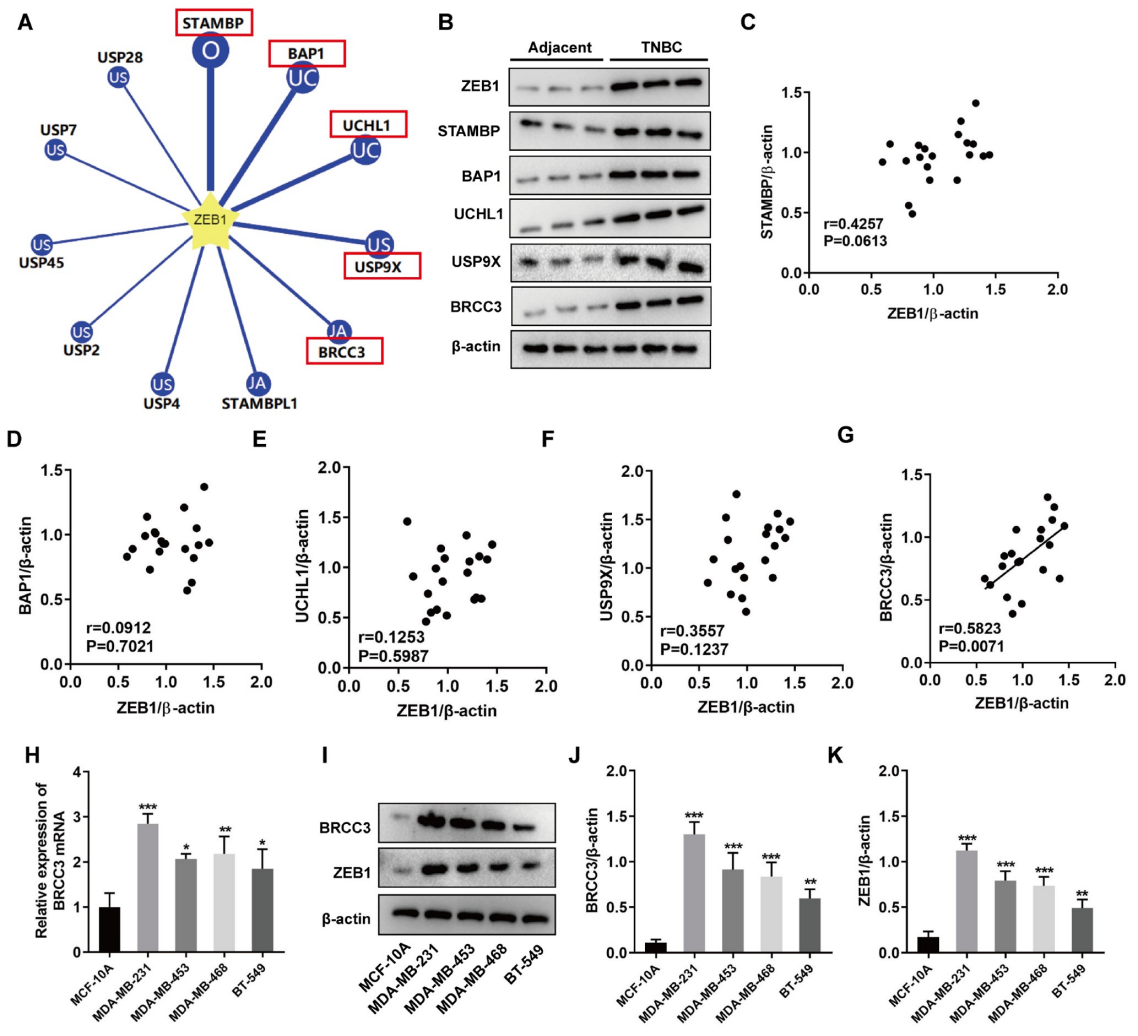
Figure 1 Screening of ZEB1-associated DUBs in TNBC (A) The online software Ubibrowser was used to screen the DUBs with the highest probability of binding to ZEB1. (B) Western blot analysis was used to detect the expressions of ZEB1, STAMBP, BAP1, UCHL1, USP9X, and BRCC3 in TNBC tissues (n=20) and adjacent nontumor tissues (n=20). (C–G) Pearson correlation analysis of the protein expressions of five DUBs and ZEB1 in 20 TNBC tissues collected in this study. (H) RT-qRCR was used to detect the mRNA levels of BRCC3 in MCF-10A, MDA-MB-231, MDA-MB-453, MDA-MB-468 and BT-549 cells. (I–K) Western blot analysis was used to detect the protein expressions of BRCC3 and ZEB1 in MCF-10A, MDA-MB-231, MDA-MB-453, MDA-MB-468 and BT-549 cells. *P<0.05, **P<0.01, and ***P<0.001 vs MCF-10A cells.

We used the online software Ubibrowser 2.0 to predict and screen five DUBs with the highest binding potential ( *i*. *e*., high prediction score) to ZEB1, including signal transducing adaptor molecule binding protein (STAMBP), BRCA1 association protein 1 (BAP1), ubiquitin carboxy-terminal hydrolase-1 (UCHL1), ubiquitin-specific peptidase 9X-linked (USP9X), and BRCC3 ( Figure 1A). The protein expression levels of ZEB1, STAMBP, BAP1, UCHL1, USP9X, and BRCC3 are obviously greater in the 20 TNBC tissue samples than in the adjacent nontumor tissue samples ( Figure 1B and Supplementary Figure S1). Next, Pearson correlation analysis was performed between the protein expression of five DUBs and ZEB1 protein expression via western blot analysis of the 20 TNBC tissues collected in this study. In addition, there are no significant correlations between the expressions of STAMBP, BAP1, UCHL1, USP9X or ZEB1 ( Figure 1C‒F). Figure 1G shows that there is a positive correlation between BRCC3 and ZEB1 protein expressions. Compared with those in MCF-10A cells, the mRNA and protein expression levels of BRCC3 in TNBC cells are significantly greater, and the BRCC3 expression level is the greatest in MDA-MB-231 cells ( Figure 1H‒J). Furthermore, Figure 1I,K show that the change in ZEB1 protein expression is consistent with that in BRCC3, and ZEB1 expression is greatest in MDA-MB-231 cells.

### Interference with BRCC3 inhibits TNBC cell proliferation, migration, invasion and EMT

**Figure 2:**
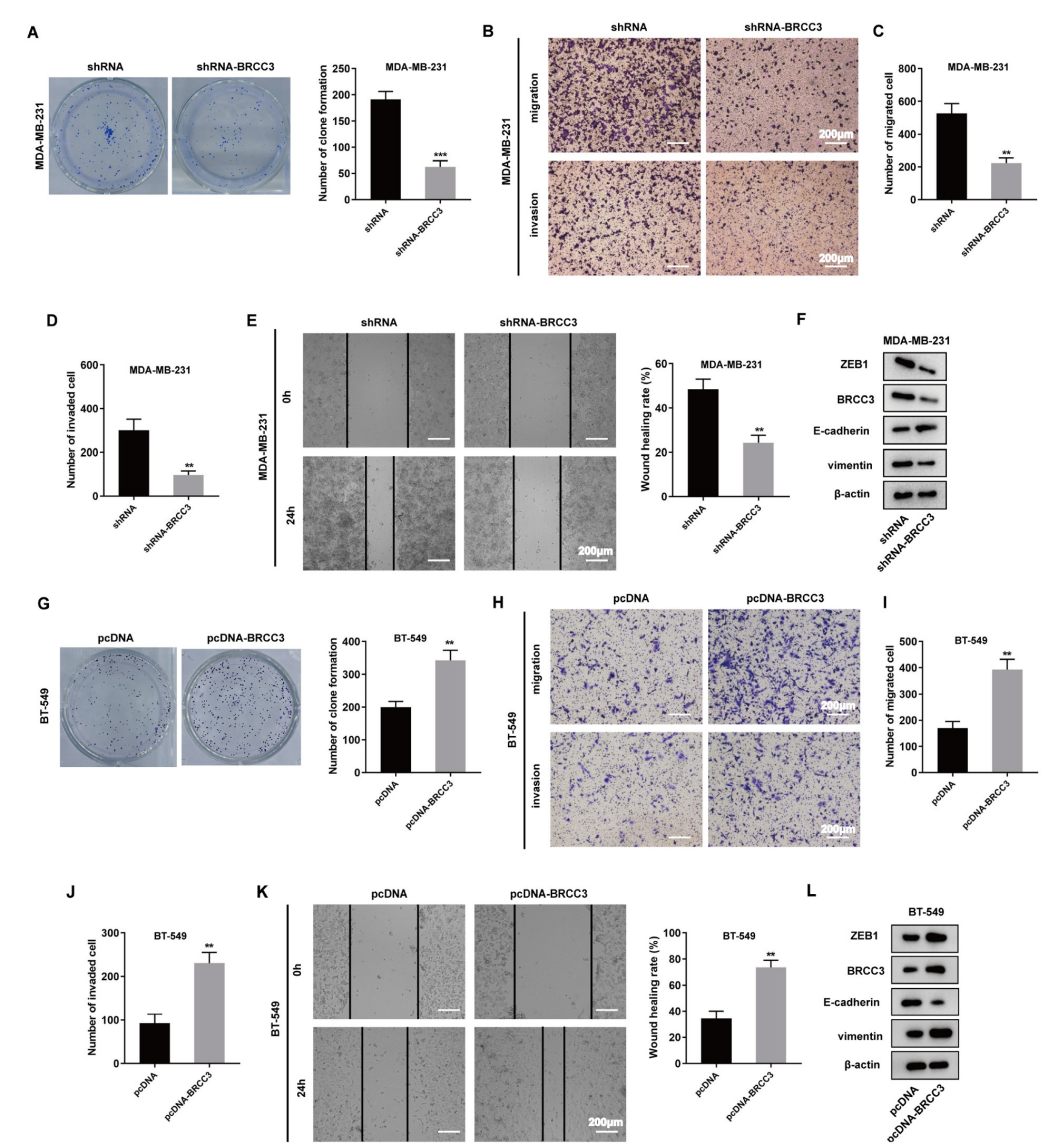
Figure 2 Interference with BRCC3 inhibits TNBC cell proliferation, migration, invasion and EMT MDA-MB-231 cells were divided into shRNA and shRNA-BRCC3 groups. (A) Cell proliferation was detected by a colony formation assay. The colony number was analyzed. ***P<0.001 vs shRNA. (B–D) Transwell assay was used to detect cell migration and invasion. **P<0.01 vs shRNA. Scale bar: 200 μm. (E) Wound healing assay was used to detect scratch mobility. **P<0.01 vs shRNA. Scale bar: 200 μm. (F) Western blot analysis was used to detect the expressions of ZEB1, BRCC3, E-cadherin and vimentin. The BT-549 cells were grouped into a pcDNA group and a pcDNA-BRCC3 group. (G) Cell proliferation was detected by a colony formation assay. The colony number was analyzed. **P<0.01 vs pcDNA. (H–J) Transwell assay was used to detect cell migration and invasion. **P<0.01 vs pcDNA. Scale bar: 200 μm. (K) Wound healing assay was used to detect scratch mobility. **P<0.01 vs pcDNA. Scale bar: 200 μm. (L) Western blot analysis was used to detect the expressions of ZEB1, BRCC3, E-cadherin and vimentin.

Based on the above findings, we next explored the influence of BRCC3 on the development of TNBC. In MDA-MB-231 and BT-549 cells, ZEB1 and BRCC3 are colocalized in the nucleus ( Supplementary Figure S2A). MDA-MB-231 cells were divided into shRNA and shRNA-BRCC3 groups. Figure 2A shows that there is less colony formation in the shRNA-BRCC3 group than in the shRNA group. Transwell assays were used to detect cell migration and invasion in each group ( Figure 2B). The number of migrated cells and the scratch mobility of cells in the shRNA-BRCC3 group are dramatically lower than those in the shRNA group ( Figure 2C,E), indicating that interference with BRCC3 has a significant inhibitory effect on the migration ability of TNBC cells. Figure 2D shows that the number of invading cells in the shRNA-BRCC3 group is significantly less than that in the shRNA group, indicating that BRCC3 knockout inhibits TNBC cell invasion. Moreover, interfering with BRCC3 increases the protein expression of the epithelial cell marker E-cadherin during EMT but downregulates the protein expressions of ZEB1, BRCC3 and the mesenchymal marker vimentin in MDA-MB-231 cells ( Figure 2F).

Next, the BT-549 cells were grouped into a pcDNA group and a pcDNA-BRCC3 group. Figure 2G shows that overexpression of BRCC3 promotes colony formation in the pcDNA-BRCC3 group, indicating that BRCC3 could regulate the clonogenic ability of TNBC cells. Transwell assays showed that overexpression of BRCC3 promotes the migration and invasion of BT-549 cells ( Figure 2H‒J). In addition, wound healing assays showed that overexpression of BRCC3 promotes cell migration ( Figure 2K). Compared with those in the pcDNA group, the protein expression levels of ZEB1, BRCC3 and vimentin are upregulated, and the protein expression level of E-cadherin is downregulated in the pcDNA-BRCC3 group ( Figure 2L). The above experiments showed that interference with BRCC3 inhibits TNBC cell proliferation, migration, invasion and EMT.

### Interference with BRCC3 promotes ZEB1 protein degradation and inhibits ZEB1 expression by increasing its ubiquitination level

**Figure 3:**
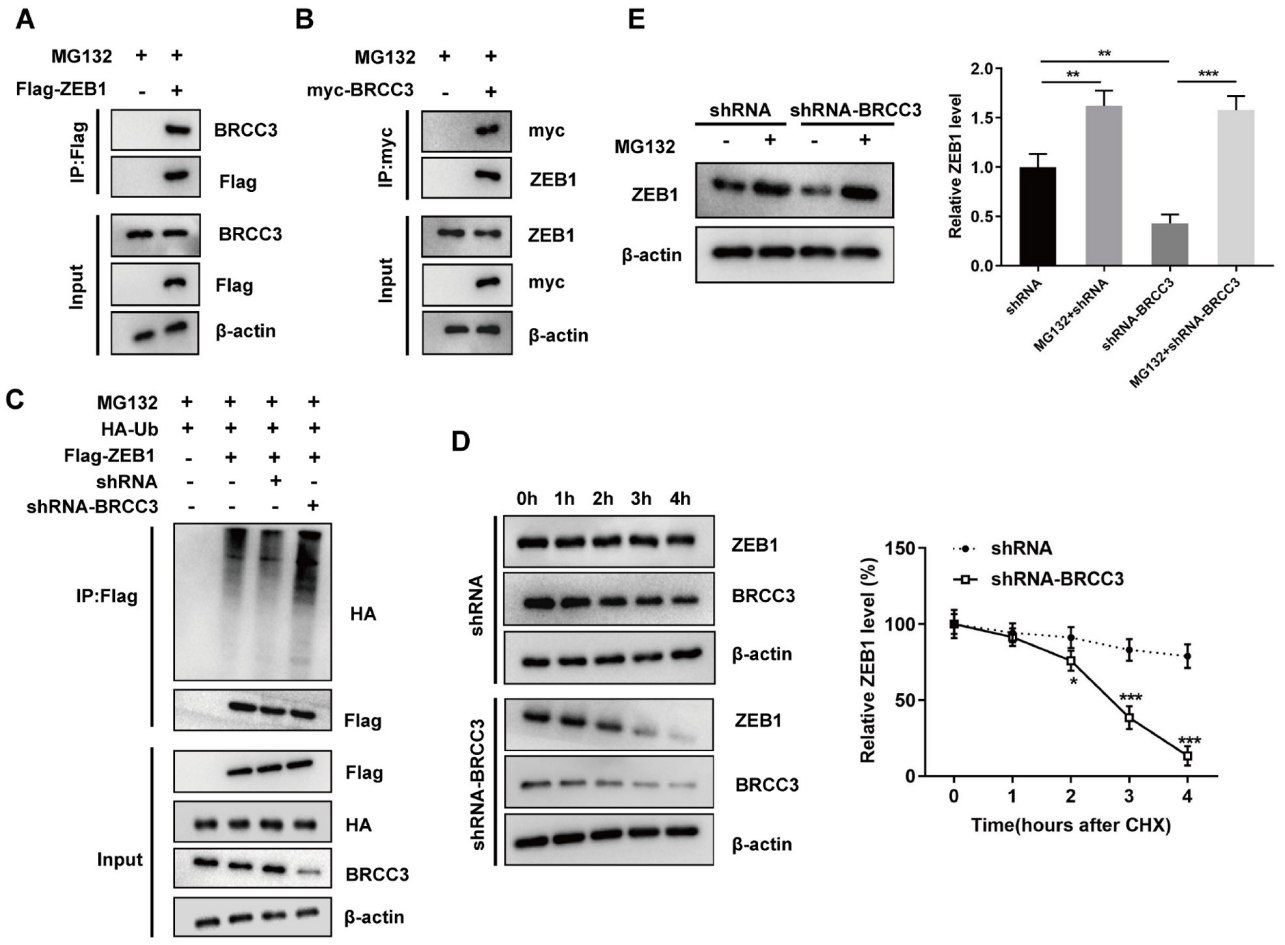
Figure 3 Interference with BRCC3 promotes ZEB1 protein degradation and inhibits ZEB1 expression by increasing its ubiquitination level (A,B) The interaction between BRCC3 and ZEB1 was verified by coimmunoprecipitation (co-IP). (C) A ubiquitination assay was conducted to confirm the ubiquitination effect of BRCC3 on ZEB1 after transfection with shRNA-BRCC3. (D) The stability of the ZEB1 protein was determined by CHX chase assay after transfection with shRNA-BRCC3. *P<0.05, ***P<0.001 vs shRNA. (E) The degradation of the ZEB1 protein after transfection with shRNA-BRCC3 was detected by MG132 treatment. **P<0.01, ***P<0.001 vs shRNA.

Since we confirmed that ZEB1 expression is regulated by BRCC3, we further verified the interaction between BRCC3 and ZEB1 using a co-IP assay. After MDA-MB-231 cells were transfected with Flag-ZEB1 and treated with MG132, BRCC3 was pulled down by co-IP with an anti-Flag antibody ( Figure 3A). After MDA-MB-231 cells were transfected with Myc-BRCC3 and treated with MG132, ZEB1 was pulled down by co-IP with an anti-Myc antibody ( Figure 3B), which indicated the interaction between BRCC3 and ZEB1. We also performed a co-IP assay in MDA-MB-231 cells and found that endogenous BRCC3 was immunoprecipitated by an anti-ZEB1 antibody ( Supplementary Figure S2B) and that endogenous ZEB1 was immunoprecipitated by an anti-BRCC3 antibody ( Supplementary Figure S2C). After interfering with BRCC3, the ubiquitination and protein degradation of ZEB1 in MDA-MB-231 cells were significantly increased ( Figure 3C,D). Furthermore, WT-BRCC3 significantly inhibited ZEB1 ubiquitination, whereas Mut-BRCC3 had no significant effect on ZEB1 ubiquitination in BT-549 cells ( Supplementary Figure S2D). We also found that interfering with BRCC3-mediated ZEB1 degradation was completely inhibited by the proteasome inhibitor MG132 ( Figure 3E).

### Interference with BRCC3 inhibits TNBC cell proliferation, migration, invasion and EMT by downregulating ZEB1

**Figure 4:**
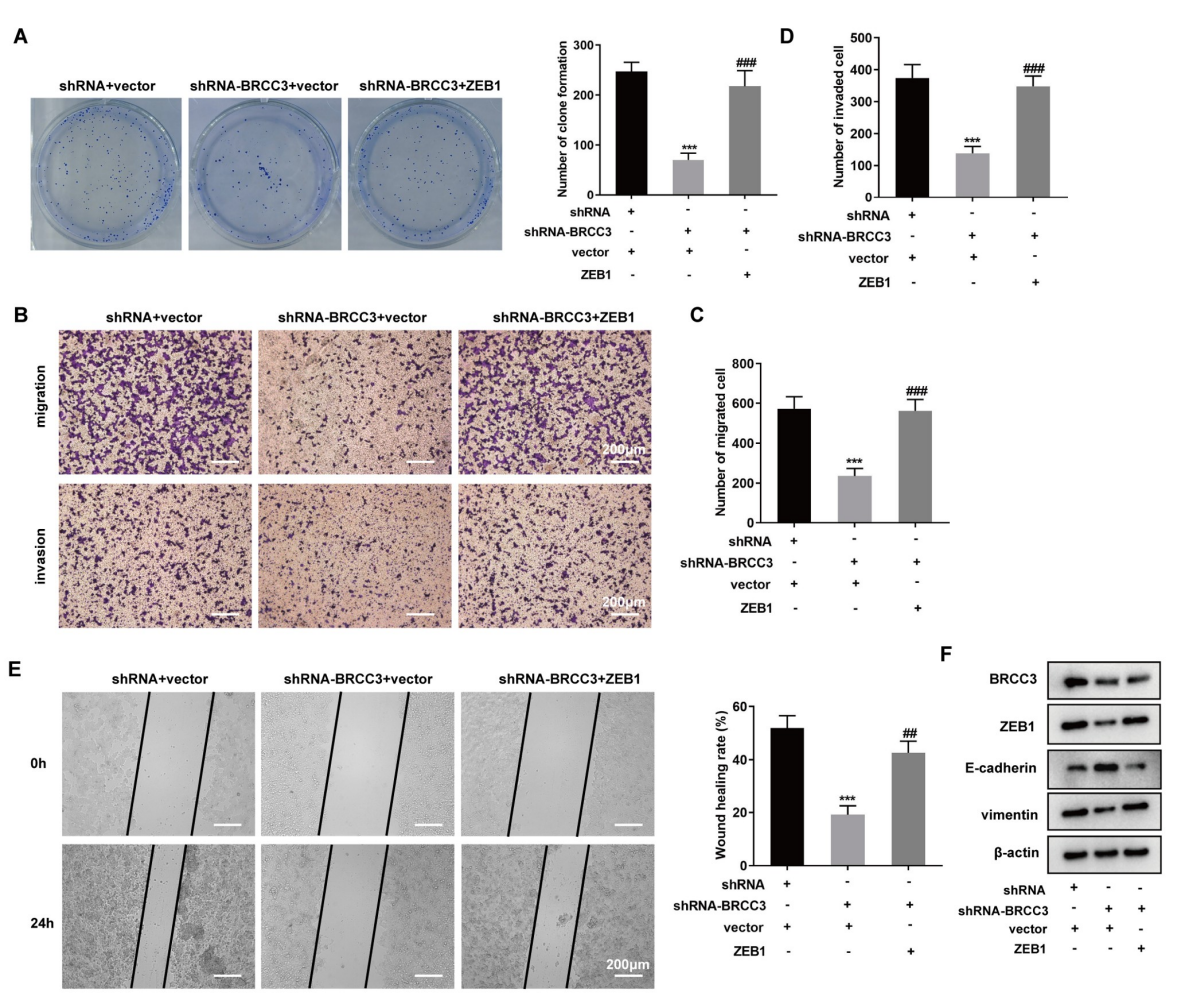
Figure 4 Interference with BRCC3 inhibits TNBC cell proliferation, migration, invasion and EMT by downregulating ZEB1 MDA-MB-231 cells were divided into shRNA+vector, shRNA-BRCC3+vector, and shRNA-BRCC3+ZEB1 groups. (A) Cell proliferation was detected by a colony formation assay. The colony number was analyzed. ***P<0.001 vs shRNA+vector, ###P<0.001 vs shRNA-BRCC3+vector. (B–D) Transwell assay was used to detect cell migration and invasion. ***P<0.001 vs shRNA+vector, ###P<0.001 vs shRNA-BRCC3+vector. Scale bar: 200 μm. (E) Wound healing assay was used to detect scratch mobility. ***P<0.001 vs shRNA+vector, ##P<0.01 vs shRNA-BRCC3+vector. Scale bar: 200 μm. (F) Western blot analysis was used to detect the expressions of BRCC3, ZEB1, E-cadherin and vimentin.

To confirm that the functions of BRCC3 depend on its ability to regulate ZEB1 expression, we cotransfected cells with the BRCC3 interference plasmid and the ZEB1 overexpression plasmid for further study. Interfering with BRCC3 inhibited colony formation, and overexpressing ZEB1 in the shRNA-BRCC3+ZEB1 group reversed this effect ( Figure 4A). Transwell assays revealed that overexpression of ZEB1 reversed the reduction in migration and invasion caused by interference with BRCC3 ( Figure 4B‒D). In addition, upregulation of ZEB1 reversed the effect of BRCC3 downregulation and improved MDA-MB-231 cell mobility ( Figure 4E). Western blot analysis showed that interference with BRCC3 upregulated E-cadherin protein expression and downregulated BRCC3, ZEB1 and vimentin protein expressions, and overexpression of ZEB1 reversed these effects ( Figure 4F). In addition, positive expression of E-cadherin and vimentin was detected by immunofluorescence microscopy in MDA-MB-231 cells. Supplementary Figure S3 shows that overexpression of ZEB1 reverses the effect of shRNA-BRCC3, downregulates the expression of E-cadherin and upregulates the expression of vimentin. The above results demonstrated that interfering with BRCC3 inhibits TNBC cell proliferation, migration, invasion and EMT by downregulating ZEB1.

### Interference with BRCC3 inhibits tumorigenesis and lung metastasis in TNBC *in vivo*

**Figure 5:**
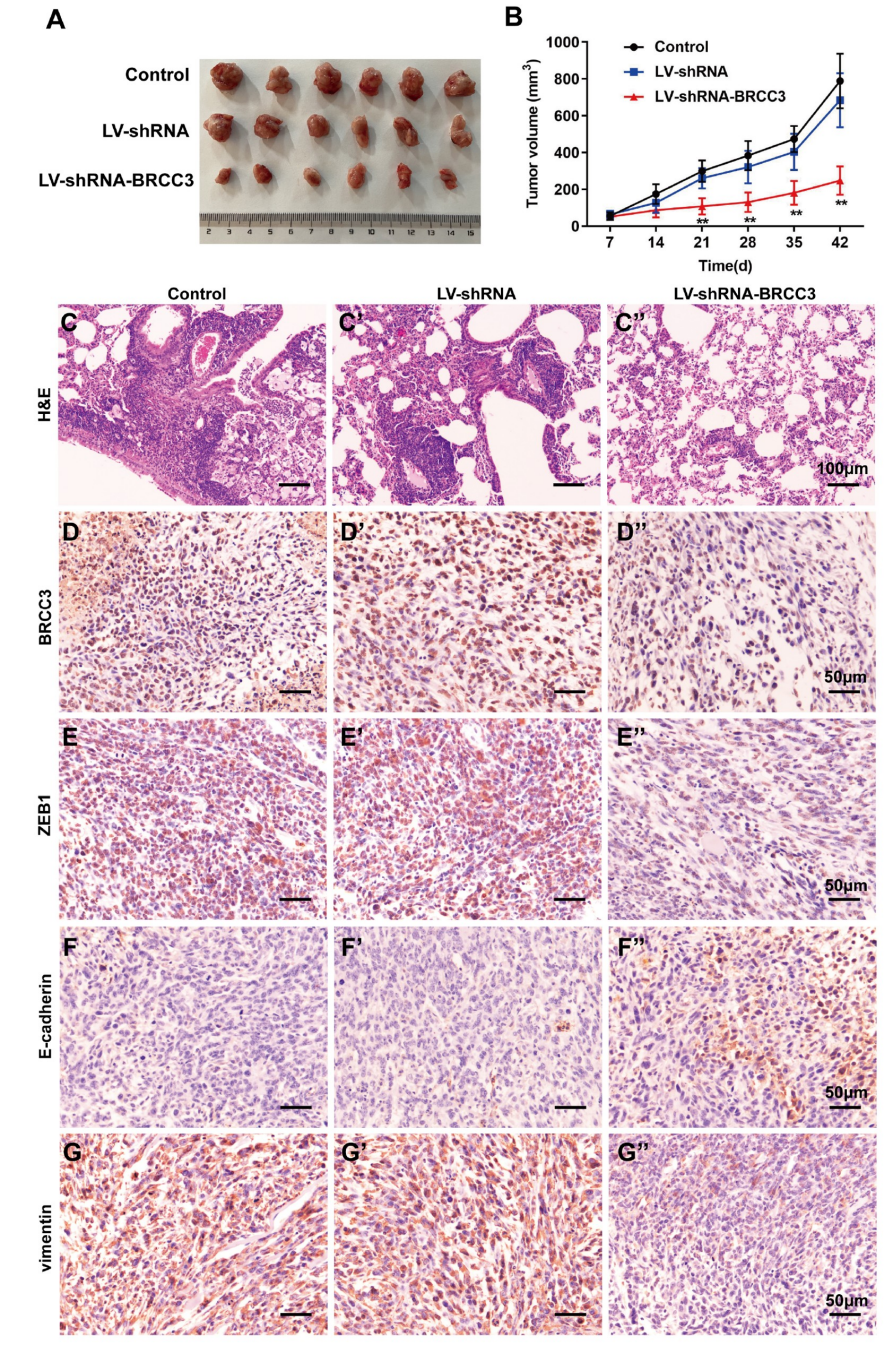
Figure 5 Interference with BRCC3 inhibits the tumorigenesis and lung metastasis of TNBC *in vivo* Mice were divided into the control, LV-shRNA, and LV-shRNA-BRCC3 groups (n=6/group). (A) The tumor tissues of each group were recorded. (B) Tumor volume was detected after 6 weeks. **P<0.01 vs LV-shRNA. (C) Lung metastasis was detected by HE staining. Scale bar: 100 μm. (D‒G) The expression levels of BRCC3, ZEB1, E-cadherin and vimentin in tumor tissues were detected via immunohistochemistry. Scale bar: 50 μm.

The changes in the transplanted tumor volume in the mice are shown in Figure 5A. After the inoculation of MDA-MB-231 cells in the control group, the subcutaneous tumors grew rapidly, and interfering with BRCC3 expression significantly inhibited tumor growth after 3 weeks ( Figure 5B). HE staining revealed deep staining of lung metastases, clear boundaries with surrounding tissues, closely arranged tumor cells and abundant capillaries in the control and LV-shRNA groups, and LV-shRNA-BRCC3 reversed these effects ( Figure 5C). Next, the expression levels of BRCC3, ZEB1, E-cadherin and vimentin in tumor tissues were detected via immunohistochemistry. We found that *BRCC3* knockdown *in vivo* upregulated E-cadherin expression and downregulated BRCC3, ZEB1 and vimentin expressions ( Figure 5D‒G). These results suggest that interference with BRCC3 inhibits EMT, TNBC cell tumorigenesis and distant metastasis.

### Interfering with BRCC3 inhibits the tumorigenesis and lung metastasis of TNBC cells *in vivo* by downregulating ZEB1

**Figure 6:**
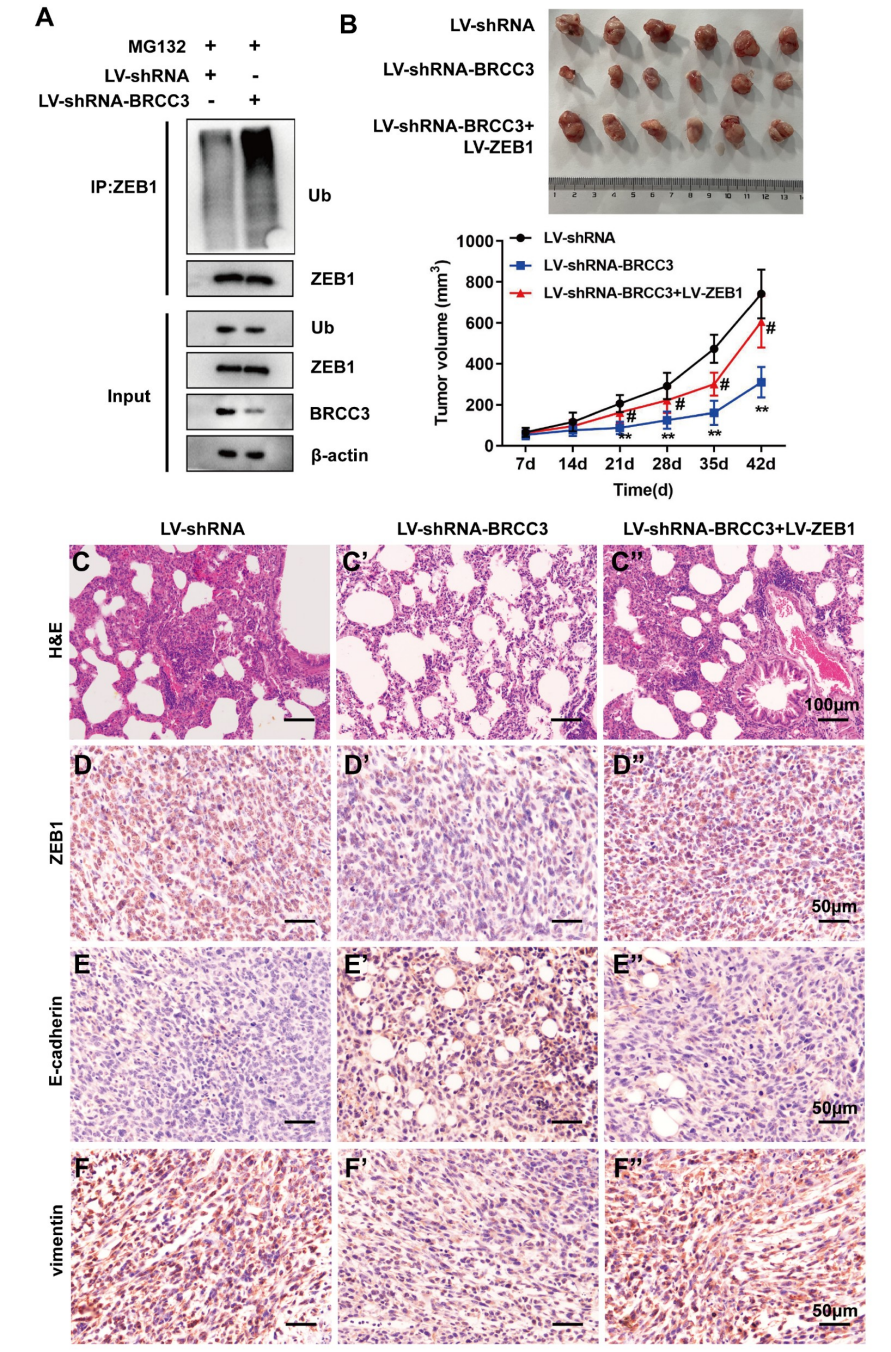
Figure 6 Interfering with BRCC3 inhibits the tumorigenesis and lung metastasis of TNBC cells *in vivo* by downregulating ZEB1 Mice were divided into LV-shRNA, LV-shRNA-BRCC3 and LV-shRNA-BRCC3+LV-ZEB1 groups (n=6/group). (A) Ubiquitination assay was performed on tumor tissues from the LV-shRNA and LV-shRNA-BRCC3 groups. (B) The tumor tissues of each group were recorded. Tumor volume was detected after 6 weeks. **P<0.01 vs LV-shRNA; #P<0.05 vs LV-shRNA-BRCC3. (C) Lung metastasis was detected by HE staining. Scale bar: 100 μm. (D‒F) The expression levels of ZEB1, E-cadherin and vimentin in tumor tissues were detected via immunohistochemistry. Scale bar: 50 μm.

To further confirm the molecular mechanism *in vivo*, we divided the mice into LV-shRNA, LV-shRNA-BRCC3, and LV-shRNA-BRCC3+LV-ZEB1 groups. Tumor tissue lysates from LV-shRNA and LV-shRNA-BRCC3 mice were subjected to IP with anti-ZEB1 antibody, followed by western blot analysis with anti-ubiquitin antibody. Mice in the LV-shRNA-BRCC3 group exhibited increased ZEB1 ubiquitination ( Figure 6A). According to the tumor tissue images and statistical analysis of the tumor volume, LV-ZEB1 reversed the effect of LV-shRNA-BRCC3 and promoted tumor growth ( Figure 6B). The results of HE staining demonstrated that LV-ZEB1 promoted lung metastasis ( Figure 6C). The immunohistochemistry results showed that BRCC3 interference upregulated E-cadherin expression and downregulated ZEB1 and vimentin expressions, and ZEB1 abolished these effects ( Figure 6D‒F).

## Discussion

The main factors leading to poor prognosis and death in TNBC patients are tumor recurrence and metastasis. Therefore, further research on the mechanism of TNBC occurrence and development is crucial for its early diagnosis and treatment. We examined the expressions of five DUBs and ZEB1 in TNBC patients and performed a correlation analysis. Only BRCC3 expression was positively correlated with ZEB1 expression, whereas STAMBP, BAP1, UCHL1, and USP9X were not significantly correlated with ZEB1 expression. BRCC3 has been reported to be a procarcinogenic factor in other cancers. For example, in nasopharyngeal carcinoma, *BRCC3* knockdown increased cell survival, attenuated DNA damage repair, and led to G2/M cell cycle arrest in radioresistant nasopharyngeal carcinoma cells [22]. In cervical cancer, interference with BRCC3 inhibited cervical cancer cell viability, invasion and migration ability [23]. However, the role of BRCC3 in TNBC metastasis has not been reported. Based on our findings, we hypothesized that BRCC3 may increase ZEB1 stability and upregulate ZEB1 expression through deubiquitination, thereby promoting TNBC cell proliferation, migration, invasion, EMT and metastasis.

It is well known that EMT is important for the invasion and metastasis of cancers, including ovarian, breast, colon, lung and liver cancers [24]. In TNBC, prominent metastatic and invasive abilities are both significant features, and EMT is closely associated with the invasion and migration of many kinds of tumor cells. Therefore, effective inhibition of EMT is highly important for the treatment of TNBC. The expressions of EMT molecular markers are tightly regulated by different transcription factors, which are known as EMT-inducing transcription factors (EMT-TFs), including Slug, Snail, Twist1, and ZEB1/2 [25]. EMT-TFs are extremely unstable proteins that are strictly controlled at the protein level by the UPS. Under pathological conditions, dysfunctional DUBs negatively affect the UPS, thereby enhancing protein stability and aggregation [26]. It was found that USP51 and constitutively photomorphogenic 9 signalosome subunit 5 promote EMT by stabilizing the expression of ZEB1, leading to increased tumor invasion and metastasis [27].

In this study, five deubiquitinating enzymes with the highest binding potential (highest score) to ZEB1, namely, STAMBP, BAP1, UCHL1, USP9X and BRCC3, were screened via the deubiquitination prediction software Ubibrowser 2.0. Knockdown of *STAMBP* could inhibit the proliferation, migration and invasion of multiple TNBC cell lines [28]. Studies have reported that BAP1 upregulates β-catenin, which further promotes TNBC tumorigenesis [29]. UCHL1 plays a role in the malignant progression of TNBC by maintaining cell stemness and promoting invasion [30]. Moreover, USP9X inhibitors inhibit TNBC cell migration, invasion and metastasis and increase cell sensitivity to cisplatin and paclitaxel [31]. Moreover, mRNA expression profiling of BRCC3 has been reported in human breast cancer cells, and exogenous BRCC3 expression is associated with delayed death and increased breast cancer cell proliferation [32]. *In vitro* experiments showed that interference with BRCC3 increases the level of ZEB1 ubiquitination and promotes ZEB1 protein degradation, thereby inhibiting ZEB1 expression.

E-cadherin and vimentin are more mature cell markers studied during the EMT process. E-cadherin is a transmembrane glycoprotein with adhesion properties that plays a role in stabilizing epithelial cell morphology and maintaining tissue structural integrity. Vimentin is generally expressed in normal mesenchymal cells and mesenchymal tumor cells, and can weaken epithelial cell adhesion and promote tumor cell invasion and migration [8]. In the present study, interfering with BRCC3 was found to inhibit the migration and invasion of TNBC cells, upregulate E-cadherin expression, and downregulate ZEB1 and vimentin expressions, and these effects were reversed by the ZEB1 overexpression plasmid. Further *in vivo* experiments confirmed that interfering with BRCC3 inhibited the EMT, tumorigenic ability and lung metastasis of TNBC cells.

In conclusion, BRCC3 promotes TNBC cell proliferation and metastasis by increasing the stability of ZEB1 under pathological conditions. In the future, molecular methods for BRCC3 interference could lead to new ideas and provide a clinical and experimental basis for TNBC treatment. Nevertheless, in this study, the clinical sample size was small and most of the experiments were performed at the protein level, leading to a lack of depth in the study. In the future, more accurate large-scale clinical studies and in-depth experimental studies should be conducted.

## Supplementary Data

Supplementary data are available at *Acta Biochimica et Biophysica Sinica* online.

## References

[1] DeSantis C, Ma J, Bryan L, Jemal A (2013). Breast cancer statistics, 2013. CA Cancer J Clin.

[2] Kwa MJ, Adams S (2018). Checkpoint inhibitors in triple‐negative breast cancer (TNBC): where to go from here. Cancer.

[3] Yin L, Duan JJ, Bian XW, Yu S (2020). Triple-negative breast cancer molecular subtyping and treatment progress. Breast Cancer Res.

[4] Kwapisz D (2021). Pembrolizumab and atezolizumab in triple-negative breast cancer. Cancer Immunol Immunother.

[5] Guestini F, McNamara KM, Ishida T, Sasano H (2016). Triple negative breast cancer chemosensitivity and chemoresistance: current advances in biomarkers indentification. Expert Opin Ther Targets.

[6] de Farias Morais HG, de Morais EF, Carlan LM, de Pontes Santos HB, da Silveira ÉJD, de Almeida Freitas R (2022). Epithelial-mesenchymal transition modulates lower lip carcinogenesis and promotes cancer progression. Arch Oral Biol.

[7] Mittal V (2018). Epithelial mesenchymal transition in tumor metastasis. Annu Rev Pathol Mech Dis.

[8] Dongre A, Weinberg RA (2019). New insights into the mechanisms of epithelial-mesenchymal transition and implications for cancer. Nat Rev Mol Cell Biol.

[9] Hirabayashi D, Yamamoto K, Maruyama A, Tomonobu N, Kinoshita R, Chen Y, Komalasari NLGY (2023). LOXL1 and LOXL4 are novel target genes of the Zn
^2+^-bound form of ZEB1 and play a crucial role in the acceleration of invasive events in triple-negative breast cancer cells. Front Oncol.

[10] Chen Y, Sumardika IW, Tomonobu N, Kinoshita R, Inoue Y, Iioka H, Mitsui Y (2019). Critical role of the MCAM-ETV4 axis triggered by extracellular S100A8/A9 in breast cancer aggressiveness. Neoplasia.

[11] Gao Y, Wang R, Liu J, Zhao K, Qian X, He X, Liu H (2022). SENP1 promotes triple-negative breast cancer invasion and metastasis via enhancing CSN5 transcription mediated by GATA1 deSUMOylation. Int J Biol Sci.

[12] Kim HY, Kim YM, Hong S (2021). DNAJB9 suppresses the metastasis of triple-negative breast cancer by promoting FBXO45-mediated degradation of ZEB1. Cell Death Dis.

[13] Luo N, Zhang K, Li X, Hu Y (2020). ZEB1 induced‐upregulation of long noncoding RNA ZEB1‐AS1 facilitates the progression of triple negative breast cancer by binding with ELAVL1 to maintain the stability of ZEB1 mRNA. J Cell Biochem.

[14] Zhang L, Yuan C, Peng J, Zhou L, Jiang Y, Lin Y, Yin W (2020). SHP-2-mediated upregulation of ZEB1 is important for PDGF-B-induced cell proliferation and metastatic phenotype in triple negative breast cancer. Front Oncol.

[15] Nabavi SF, Atanasov AG, Khan H, Barreca D, Triombetta D, Testai L, Sureda A (2018). Targeting ubiquitin-proteasome pathway by natural, in particular polyphenols, anticancer agents: lessons learned from clinical trials. Cancer Lett.

[16] Wang Q, Ma S, Song N, Li X, Liu L, Yang S, Ding X (2016). Stabilization of histone demethylase PHF8 by USP7 promotes breast carcinogenesis. J Clin Invest.

[17] Li X, Yuan J, Song C, Lei Y, Xu J, Zhang G, Wang W (2021). Deubiquitinase USP39 and E3 ligase TRIM26 balance the level of ZEB1 ubiquitination and thereby determine the progression of hepatocellular carcinoma. Cell Death Differ.

[18] Ye D, Wang S, Huang Y, Wang X, Chi P (2021). USP43 directly regulates ZEB1 protein, mediating proliferation and metastasis of colorectal cancer. J Cancer.

[19] Liu Y, Zeng S, Zhang W, Li J, Yin Y, Zhuang Y, Zhou J (2023). USP51/ZEB1/ACTA2 axis promotes mesenchymal phenotype in gastric cancer and is associated with low cohesion characteristics. Pharmacol Res.

[20] Li J, Xiao X, Wang H, Wang W, Ou Y, Wang Z, Jiang H (2022). CDK4/6-USP51 axis regulates lung adenocarcinoma metastasis through ZEB1. Cancer Gene Ther.

[21] Mrohs D, Rybarski M, Andriske M, Bohne P, Mark MD, Lübbert H, Zhu XR (2023). Additional polgD257A mutation (mutator) does not influence dopaminergic neurodegeneration in aged parkin-deficient mice. Aging Pathobiol Ther.

[22] Tu Z, Xu B, Qu C, Tao Y, Chen C, Hua W, Feng G (2015). BRCC3 acts as a prognostic marker in nasopharyngeal carcinoma patients treated with radiotherapy and mediates radiation resistance
*in vitro*. Radiat Oncol.

[23] Zhang F, Zhou Q (2018). Knockdown of BRCC3 exerts an anti‑tumor effect on cervical cancer
*in vitro*. Mol Med Report.

[24] Guarino M (2007). Epithelial-mesenchymal transition and tumour invasion. Int J Biochem Cell Biol.

[25] Inoue Y, Itoh Y, Sato K, Kawasaki F, Sumita C, Tanaka T, Morishita D (2016). Regulation of epithelial-mesenchymal transition by E3 ubiquitin ligases and deubiquitinase in cancer. Curr Cancer Drug Targets.

[26] Antao AM, Tyagi A, Kim KS, Ramakrishna S (2020). Advances in deubiquitinating enzyme inhibition and applications in cancer therapeutics. Cancers.

[27] Zhang S, Hong Z, Chai Y, Liu Z, Du Y, Li Q, Liu Q (2017). CSN5 promotes renal cell carcinoma metastasis and EMT by inhibiting ZEB1 degradation. Biochem Biophys Res Commun.

[28] Yang Q, Yan D, Zou C, Xue Q, Lin S, Huang Q, Li X (2022). The deubiquitinating enzyme STAMBP is a newly discovered driver of triple-negative breast cancer progression that maintains RAI14 protein stability. Exp Mol Med.

[29] Tang J, Li Y, Sang Y, Yu B, Lv D, Zhang W, Feng H (2018). LncRNA PVT1 regulates triple-negative breast cancer through KLF5/beta-catenin signaling. Oncogene.

[30] Tian C, Liu Y, Liu Y, Hu P, Xie S, Guo Y, Wang H (2022). UCHL1 promotes cancer stemness in triple-negative breast cancer. Pathol Res Pract.

[31] Guan T, Yang X, Liang H, Chen J, Chen Y, Zhu Y, Liu T (2022). Deubiquitinating enzyme USP9X regulates metastasis and chemoresistance in triple‐negative breast cancer by stabilizing Snail1. J Cell Physiol.

[32] Boudreau HE, Broustas CG, Gokhale PC, Kumar D, Mewani RR, Rone JD, Haddad BR,
*et al*. Expression of BRCC3, a novel cell cycle regulated molecule, is associated with increased phospho-ERK and cell proliferation.
Int J Mol Med 2007, 19: 29–39. https://pubmed.ncbi.nlm.nih.gov/17143545/.

